# The Influence of Microwave Sterilization on the Ultrastructure, Permeability of Cell Membrane and Expression of Proteins of *Bacillus Cereus*

**DOI:** 10.3389/fmicb.2018.01870

**Published:** 2018-09-04

**Authors:** Jin-Xuan Cao, Fang Wang, Xuan Li, Yang-Ying Sun, Ying Wang, Chang-Rong Ou, Xing-Feng Shao, Dao-Dong Pan, Dao-Ying Wang

**Affiliations:** ^1^Key Laboratory of Animal Protein Food Processing Technology of Zhejiang Province, Ningbo University, Ningbo, China; ^2^Institute of Agricultural Products Processing, Jiangsu Academy of Agricultural Sciences, Nanjing, China

**Keywords:** *Bacillus cereus*, microwave, morphology, the permeability of membrane, proteomics

## Abstract

*Bacillus cereus* was isolated from ready-to-serve brine goose, identified by 16S rRNA gene sequencing analysis and treated with a commercial microwave sterilization condition (a power of 1,800 W at 85°C for 5 min). The influence of microwaves on the morphology, the permeability of membrane and the expression of total bacterial proteins was observed. Microwave induced the clean of bacterial nuclear chromatin, increased the permeability and disrupted the integrity of membrane. Twenty-three proteins including 18 expressed down-regulated proteins and 5 expressed up-regulated proteins were identified by HPLC-MS/MS in the samples treated with microwave. The frequencies of proteins changed after microwaves treatment were labeled as 39.13% (synthesis and metabolism of amino acid or proteins), 21.74% (carbohydrate metabolism), 8.70% (anti-oxidant and acetyl Co-A synthesis), and 4.35% (the catalyst of catabolism of bacterial acetoin, ethanol metabolism, glyoxylate pathway, butyrate synthesis and detoxification activity), respectively. This study indicates that microwaves result in the inactivation of *Bacillus cereus* by cleaning nuclear chromatin, disrupting cell membrane and disordering the expression of proteins.

## Introduction

*Bacillus cereus* (*B. cereus*) is an aerobic spore former commonly in raw meat, ready-to-serve meats, meat products, and meat additives (Gueven et al., 2006). *B. cereus* causes two types of food-borne illnesses: one type is characterized by nausea and vomiting; another type is manifested primarily by abdominal cramps and diarrhea (Granum and Lund, 1997). The population of *B. cereus* increases by two times from 20 min to 3 h at 30°C in the different food products (Mikkola, 2006). The presence of *B. cereus* in ready-to-serve meat products that have not been subjected to sterilization treatment is a public health concern. Food borne illnesses from *B. cereus* occurs due to the survival of bacterial endospores when food is improperly cooked or refrigerated (McKillip, 2000). The growth of *B. cereus* results in the production of enter toxins which is highly resistant to heat and acids (pH levels between 2 and 11; Ehling-Schulz et al., 2004).

There have been various attempts to eliminate or control the growth of *B. cereus* by natural active chemicals, food additives, irradiation, and high hydrostatic pressure (Lado and Yousef, 2002). The injury and lethality of heat treatment of *B. cereus* strain have been analyzed. As a new treatment way, the use of microwave radiation became popularly in muscle foods, particularly in the case of ready-to-serve meat products. It took a great convenience for all of households to avoid destroying the nutrient and sensory quality of food compared with conventional thermal treatments in the past decades (Chandrasekaran et al., 2013). Some results have been reported with regard to the sterilization of microwaves on microorganisms (Yaghmaee and Durance, 2005). However, the effect of microwave on *B. cereus* is far from being understood.

Some reports have focused on the influence of microwave on cell membrane, cell wall, soluble chemical oxygen demand (Zhou et al., 2010), the inactivation of enzymes, and functional disorder (Shahin et al., 2015). Vela and Wu (1979) thought that the destruction of microorganisms was mainly due to a thermal effect of microwave exposure. Kozempel et al. (1998) observed that the destruction of microorganisms by microwave at low temperatures (below 40°C) was more effective than the thermal destruction. Although the effect of microwave treatment on overall cell function has been reported preliminarily, the mechanism of the inactivation of *B. cereus* has received scant research attention. In an effort to further understand how microwave treatment influences the sterilization of *B. cereus*, this study was conducted to determine the influence of microwaves treatment on the morphology, the permeability of cell membrane and the expression of total bacterial proteins.

## Materials and methods

### Isolation of *B. cereus* from meat product

Ready-to-serve brine geese were purchased from local supermarket. Then the product was put on a tray covered with plastic wrap (oxygen, carbon dioxide, and water vapor transmission rates of the film were 14,483 cm^3^/(m^2^ × 24 h × atm), 63,683 cm^3^/(m^2^ × 24 h × atm) and 54 g/(m^2^ × 24 h), respectively) at 25°C for 14 d to induce spoilage. For spoilage microorganisms, 25 g of samples was added into 225 ml of 0.85% sterile physiological saline in an Erlenmeyer flask; then the flask was incubated in the shaking incubator at 230 rpm for 1 h at room temperature. *B. cereus* was isolated as described by Tallent et al. (2012) with slight modification. Briefly, 1 ml of sample was diluted with 9 ml of 0.85% sterile physiological saline to be an initial dilution (10^−1^). Then the culture was decimally diluted; 0.1 ml of the appropriate dilution (10^−4^, 10^−5^, and 10^−6^) was spread-plated on Mannitol-Yolk-Polymyxin agar (No.1.05267, Merck, Germany). Microorganisms were incubated at 30°C for 24 h until colony development. The strains were purified by streaking selective medium (Mannitol-Yolk-Polymyxin agar) to obtain well-separated single colonies after incubation at 30°C for 24 h. The purified strains were affirmed according to colony morphology after growing on Mannitol-Yolk-Polymyxin agar. Pure strains were kept as a glycerol stock at −80°C.

### Identification of the isolate by 16S rRNA sequence analysis

DNAeasy Tissue kit (Qiagen, Valencia, CA, USA) was used to extract DNA from the cultures of purified isolates, according to the manufacturer's instructions. The extracted DNA was stored at −80°C for further use. To further identify the strains, polymerase chain reaction (PCR) was performed to amplify part of the bacterial 16S rRNA gene. PCR amplification was performed in a thermocycler (Gene Technologies, Braintree, United Kingdom) in a total volume of 50 μl, containing 1.0 μl (40 ng) of DNA, 10 μl of 5X Phire reaction buffer, 200 μM of each dNTP (1.0 μl), 10 μM of each primer (2.0 μl), 0.5 μl of Phire Hot Start II polymerase (Thermo Scientific, USA), and nuclease-free water (Promega) up to 50 μl. Primers for 16S rRNA gene amplification were 27F (5′-AGAGTTTGATCCTGGCTCAG-3′) and 1492R (5′-CTACGGCTACCTTGTTACGA-3′). The procedure for PCR amplification was performed according to the following program: initial denaturation at 95°C for 5 min; 35 cycles of 95°C for 30 s, 58°C for 30 s and 72°C for 90 s; a final extension at 72°C for 7 min. The PCR products were confirmed by electrophoresis on an agarose gel and visualized under UV light after SYBRSafe (Invitrogen) staining. PCR products were purified with High Pure PCR Cleanup Micro Kit (Roche Applied Science, USA) and sent for sequencing. Sequencing was performed by Microbe Sequencing Facility (Nanjing genscript biotechnology co., Nanjing, China). The nucleotide sequences of 16S rRNA of the isolate were analyzed and determined by the BLAST program on the NCBI website (http://www.ncbi.nlm.nih.gov) as Supplementary Material showing.

### Microwave treatment of *B. cereus*

The pure bacterial strain was incubated at 30°C, shaken at 130 rpm for 12 h to logarithmic phase, and then centrifuged at 5,000 g for 20 min. The precipitation was washed twice with 0.85% sterile saline solution; then the strains were re-suspended in 0.85% sterile saline solution containing culture in exponential growth phase of 10^7^ CFU ml^−1^. The glass container with 150 ml of bacterial suspension was placed in the center of an oven (YQ5G-03, Yongqing corp., Nanjing, China) and exposed to microwave. The temperature of suspension was accurately determined by an infrared sensor at the top of the microwave equipment. The condition of microwave was set with a commercial 2450 MHz microwave sterilization (a power of 1,800 W at 85°C for 5 min). In an interval-working way, when temperature was above 85°C, the sterilization was interrupted. The temperature was monitored by an infrared probe. The total time when samples were exposed to microwave was 5 min. Non-treated suspension was used as the control.

### Scanning electron microscopy (SEM)

SEM was used to observe the morphological changes according to the method as described by Bajpai et al. (2009) with modification. The suspension was centrifuged at 3,000 g for 5 min and washed twice with 0.1 M sodium phosphate buffer solution (pH 7.2). The precipitation was fixed in 2.5% glutaraldehyde at 4°C for 4 h. The samples were dehydrated in a sequential graded ethanol (30, 50, 70, 80, 90, and 100%); then the ethanol was then replaced by pure tertiary butyl alcohol. Eventually, all samples were dried on an Alpha 1–4 freeze drying machine (LDplus; Christ). The dried strains were sputter-coated with gold in an ion coater and observed by a S-3400N scanning electron microscope (Hitachi corp., Japan).

### Transmission electron microscopy (TEM)

Transverse micro-structural changes were analyzed by TEM according to the method of Hartmann et al. (2010). The suspensions were centrifuged for 20 min at 8,000 g. The resultant pellets were re-suspended in 0.1 M sodium phosphate buffer (pH 7.2) including 4% glutaraldehyde solution. After fixation for 12 h, the strains were washed twice with the same buffer and then post-fixed in 1% OsO_4_ in 0.1 M sodium phosphate buffer for 2 h at room temperature. After dehydrating in a graded series of ethanol, the strains were washed with acetone, embedded in Epon 812 and then observed using a JEM-1230 transmission electron microscope (Hitachi corp., Japan).

### The determination of permeability of cell membrane

The determination of transparent rate of cell membrane with the staining of propidium iodide was performed according to the procedure of Pagán and Mackey (2000). Fluorescence was measured with a 96-Well Plate Reader M200 (Tecan, Austria); the excitation wavelength was set at 495 nm; the emission wavelength was set at 615 nm. The slit width was 10 nm. Fluorescence data for strains were normalized against OD680 (background fluorescence). The transparent rate was expressed as the fluorescence values obtained from samples. The quantification of the release of nuclear material was completed according to Aronsson et al. (2005). After treating by microwave, the suspension was centrifuged at 1,500 g at 4°C for 20 min with a refrigerated centrifuge (Hunan Xiangyi Laboratory Instrument Development Co., Changsha, China). The content of nucleic acid of the supernatants was directly measured at 260 nm by detecting the UV absorbance with a 96-Well Plate Reader M200 (Tecan, Austria). The permeability of cell membrane was presented using the leakage of nucleic acid of the supernatants. Each experiment was performed in triplicate.

### Proteomic analysis of total bacterial proteins

#### Two-dimensional gel electrophoresis (2-DE)

The total bacterial proteins were extracted according to the method as described by Duffes et al. (2000) with minor modification. Bacterial suspensions were harvested by centrifuging at 9,500 g for 10 min. The cells were washed thrice with sodium phosphate buffer (pH 7.2). The cells were suspended with a lysis buffer containing 7 M urea, 2 M thiourea, 40 mM Tris-HCl (pH 6.8), 65 mM DL-dithiothreitol, 2% V/V IPG buffer, 4% W/V 3-(3-cholamido-propyl)-dimethylammonio-1-propanesulfonate, and 1% protease inhibitor cocktail. The lysate was centrifuged at 12,000 g for 30 min. The supernatant containing intracellular soluble proteins was collected and stored at −20°C in sealed micro-centrifuge tubes for further research. The 2-DE was carried out to study the differential expression of proteins. Isoelectric focusing (IEF) (Bio-rad corp., PA) was performed using nonlinear gradient IPG strips (17 cm) of pH 4~7 and a programmed voltage gradient. The IPG strips were rehydrated with samples at 50 V for 13 h, subsequently; IEF was desalted at 250 V for 30 min, 1,000 V for 1 h, 10,000 V for 5 h to reach a total of ~6,000 V ^*^ h, and then run at 500 V for 5 h. After finishing the IEF operation, the strips were equilibrated by gentle shaking in two steps for 15 min each in equilibration buffer 1 [1% DTT, 50 mM Tris-HCl (pH 6.8), 6 M urea, 30% glycerol, and 2% sodium dodecyl sulfate (SDS)] and equilibration buffer 2 [2.5% iodacetamide, 50 mM Tris-HCl (pH 6.8), 6 M urea, 30% glycerol and 2% SDS]. Afterwards, the IPG strips were loaded on 12.5% acrylamide gels in the second dimension with 1 W for 1.5 h and 15 W for 8 h at 15°C with EttanTM DALT SIX System (GE Corp., Switzerland). Separated proteins were visualized by silver diamine staining.

#### Visualization

Silver-stained 2-DE gels were scanned by a GS-800 imaging densitometer (Bio-rad corp., PA). The digitized images were analyzed by the ImageMaster software package (PDQuest 8.0, Bio-rad corp., PA).

#### In-gel trytic digestion

Protein spots were cut off from the preparative gels and destained with 100 mM NH_4_HCO_3_ in 30% acetonitrile for 30 min twice to thrice. After removing the destaining buffer, gel pieces were lyophilized and rehydrated in 30 μl of 50 mM NH_4_HCO_3_ containing 10 ng trypsin. After digestion at 37°C overnight, the peptides were extracted with 50–100 μl of 5% trifluoroacetic acid (TFA) at 40°C for 1 h, re-extracted with the same volume of 50% acetonitrile and 2.5% TFA solution at 30°C for 1 h and re-extracted by ultrasonic with 50 μl acetonitrile once again. Extracts were pooled together and lyophilized for further analysis. Matrix-Assisted Laser Desorption/Ionization mass spectrometry with automated tandem time of flight fragmentation of selected ions (MALD/I-TOF/TOF) of digested proteins was acquired with a 4700 Proteomics Analyzer mass spectrometer (Applied Biosystems, MA) in the positive reflectron mode with a 200 HZ Nd-YAG 355 nm laser. Spectra were obtained by averaging 1000 acquired spectra in the MS mode or 2500 in the MS/MS mode. Collision induced dissociation (CID) with air as the collision gas at ~1 × 10^−6^ Torr and a 1 keV acceleration voltage was used for obtaining the mass spectrometer/mass spectrometer spectra (MS/MS) of selected peptides. All MS/MS spectra were searched using the MASCOT search engine. The MS/MS of selected peptides were combined and queried against primary sequence databases by MASCOT search engine 2.2 (Matrix Science, Ltd, London, UK). A minimum MASCOT score of 50 was used to identify peptides. Peak list of gel spots were searched against the *B. cereus* database release 15 with the following criteria: precursor tolerance and the product ion tolerances were at 100 ppm and ± 0.5 Da, respectively. Fixed modification was set as Carbamidomethylation (C), Modification of methionine acetylation (Protein N-term), Deamidated (NQ), and Oxidation (M).

#### Identification of protein spots and assignment to functional classes

The identified proteins were assigned a Gene Ontology (GO) (http://www.geneontology.org) term according to their molecular function and grouped into functional categories using the “GO-MIPS funcat conversion table” (http://www.geneontology.org/external2go/mips2go) set up at the Munich Information Center for Protein Sequences (MIPS Institute). In some cases, where the GO term assigned to the protein appeared too broad, proteins were assigned to MIPS funcats (http://www.mips.gsf.de/projects/funcat) according to their roles described in the literature.

### Statistical analysis

The assays in the present study were performed in triplicate. The data on the transparent rate of bacterial membranes, optical density at 260 nm and the density of protein spots before and after microwave treatment were evaluated via Student's *t*-test of SAS 8.0 software (SAS Institute Inc., Cary, NC). The significance of difference was set as *p* < 0.05.

## Results and discussion

### Identification of the purified strains

The colony morphology of the purified strains which grew on Mannitol-Yolk-Polymyxin agar showed uniform individual pink colonies. DNA of the strains was extracted and further identified by PCR and sequencing analysis. According to the results of BLAST program, the isolate was identified as *B. cereus*, and the GenBank number was KU312197.1.

### Influence of microwave treatment on the morphological changes of the cell membrane of *B. cereus*

**Figure 1:**
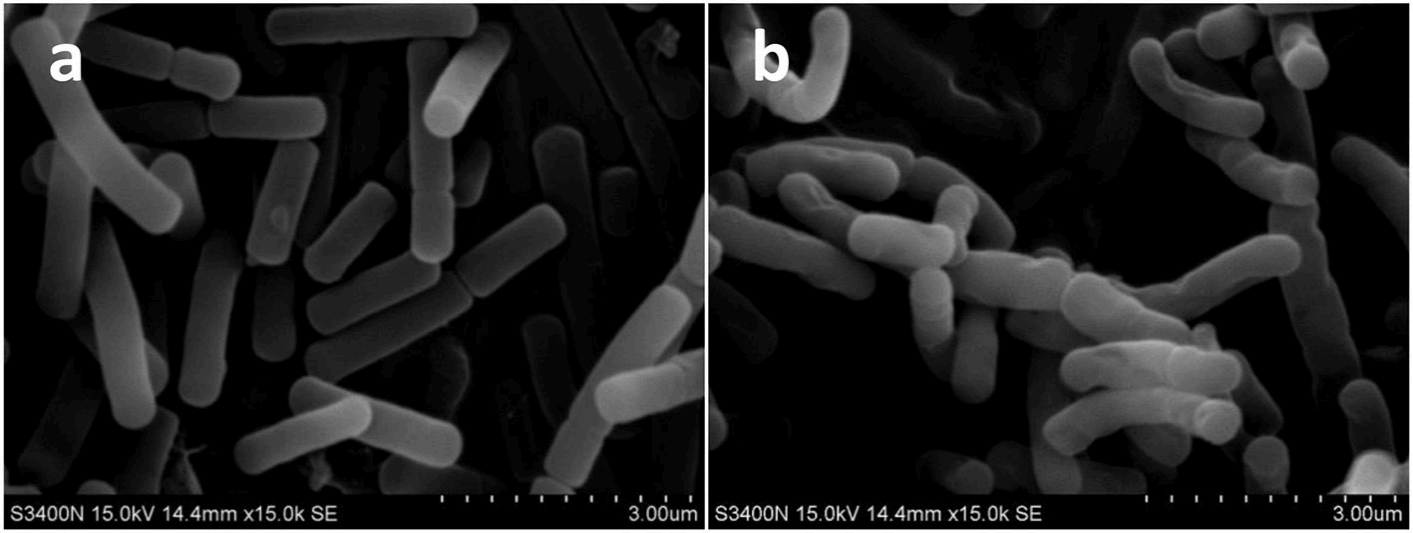
The scanning electron microscope (SEM) photography of *Bacillus cereus* in control group (× 1,500) **(A)** and microwave treatments group (× 1,500) **(B)**, respectively.

SEM analysis was performed to observe the morphological changes of *B. cereus*. As was shown in Figure 1, the control presented smooth cell membrane of *B. cereus* (1a). In contrast, samples treated with microwave revealed severe morphological destruction (1b). In addition, the surface of *B. cereus* induced by microwave was wrinkled, compared with control. This finding confirmed a previous report by Kim et al. (2008), in which high power microwave destroyed the membrane and wall of *B. cereus*.

### Influence of microwave treatment on the nuclear chromatin of *B. cereus*

**Figure 2:**
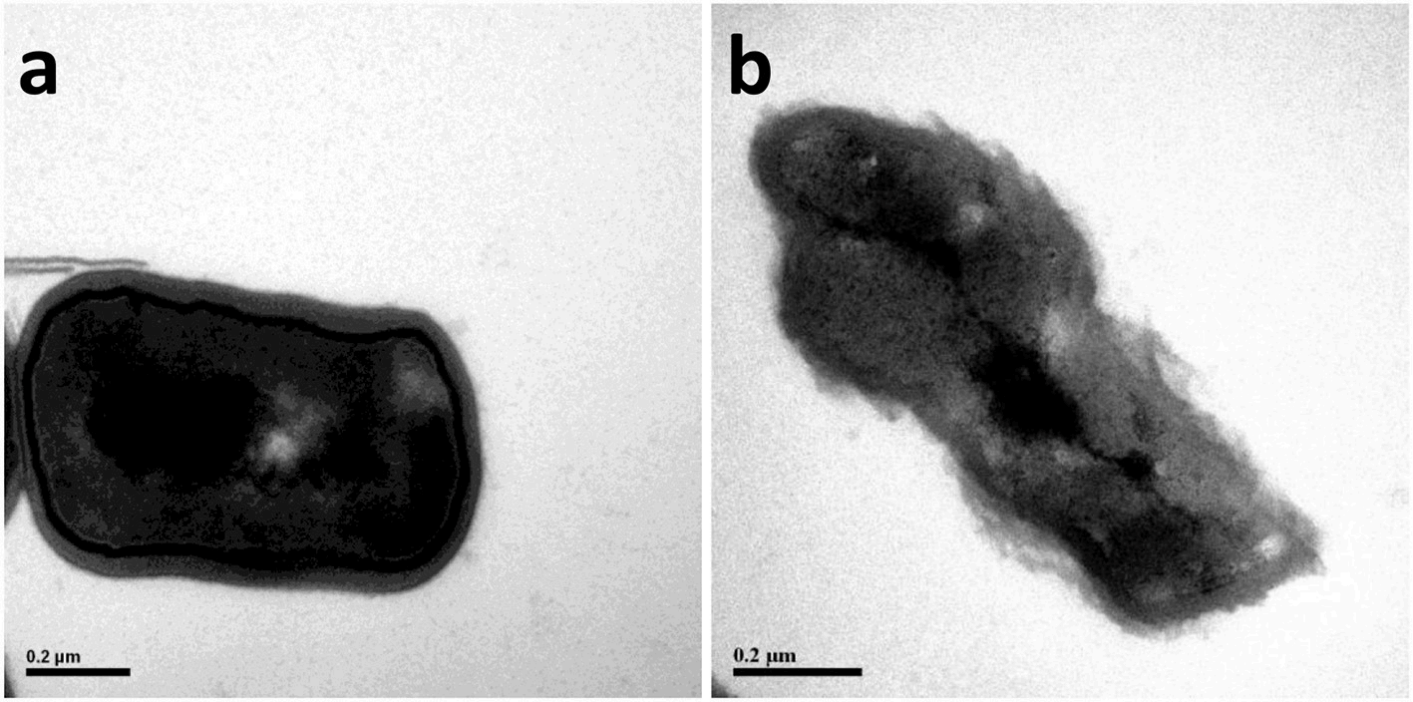
The transmission electron microscope (TEM) photography of *Bacillus cereus* in control group (× 2,500) **(A)** and microwave treatments group (× 2,500) **(B)**, respectively.

The nuclear chromatin of *B. cereus* before and after microwave treatment was observed by TEM (Figure 2). The TEM images of high electron-dense indicated that the bacterial nuclear chromatin of control samples was very uniform; nuclear cores of control samples were clear and structurally intact; the edge of bacterial membrane of control samples was smooth and normal (Figure 2A). In contrast, the low electron-dense residual nuclear chromatin and blank areas of microwave treated samples suggested that the bacterial nuclear chromatin was cleaned partly (Figure 2B).

The decrease of bacterial nuclear chromatin could be attributed to the disruption of cell membrane by microwave treatment. In addition, microwave treatment has been demonstrated to induce the aggregation of nuclear chromatin and the appearance of lower-density blank areas (Ikarashi et al., 1986).

### The effect of microwave treatment on the permeability of bacterial membrane

The effect of microwave treatment on the transparent rate and the release of intracellular components of *B. cereus* was listed in Table 1. The transparent rate of *B. cereus* treated with microwave (68.63%) was higher than that of the control (7.13%) (*p* < 0.001). The optical density at 260 nm in the bacterial suspension increased significantly with microwave treatment (*p* < 0.001); it indicated that the nuclear components were released from cytoplasm (Gedikli et al., 2008).

**Table 1:** The transparent rate of bacterial membranes and optical density at 260 nm before and after microwave treatment.

	**Control group**	**Microwave treating group**
Transparent rate(%)	7.13 ± 0.26^b^	68.63 ± 0.73^a^
Optical density at 260 nm	< 0.01^b^	0.27 ± 0.06^a^

a−b *Identical letters in the same row indicate that there was no significant difference (p > 0.05)*.

The results of microwave treatment on the transparent rate of *B. cereus* coincided with our morphological results by SEM analysis. The increase of optical density at 260 nm in the bacterial suspension explained the elimination of bacterial nuclear chromatin treated by microwave in TEM results. These results were similar to the reports by Shin and Pyun (1997), who found that the optical density at 260 nm in the bacterial suspension increased to above 0.4 and 0.6 after treating with continuous microwave and pulse microwave respectively at 50°C for 20 min for *Lactobacillus plantarum*. Furthermore, Diao et al. (2014) implied that the destruction of cell membranes led to the leakage of intracellular components, the impairment of enzyme systems and the death of microorganisms. In our study, the leakage of intracellular components from *B. cereus* could contribute to the higher optical density OD280 in microwave treatment samples than those in the control.

### The effect of microwave treatment on the expression of total bacterial proteins

**Figure 3:**
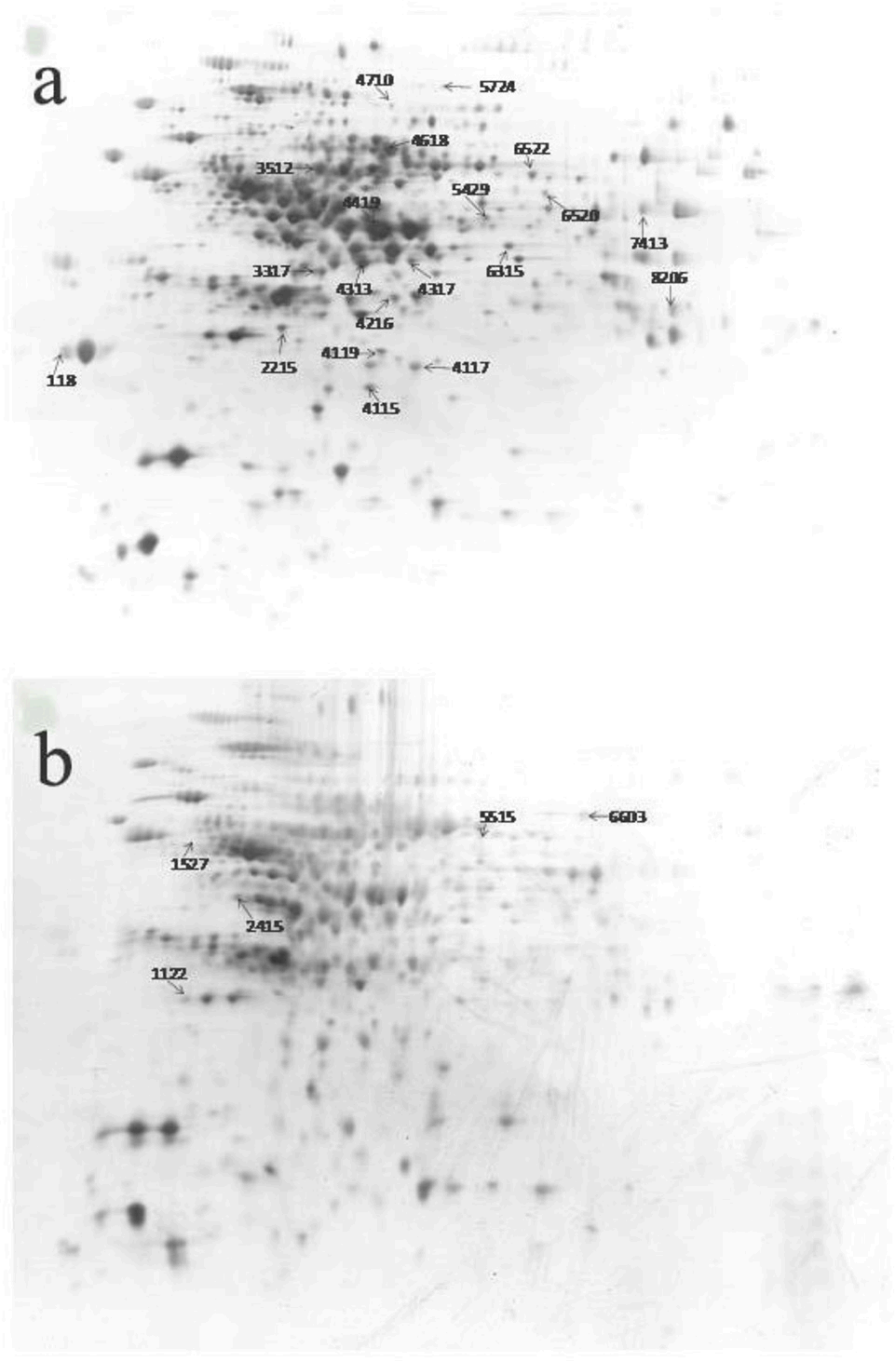
2-DE pattern of total proteins of *Bacillus cereus* in control group **(A)** and microwave treatments group **(B)**, respectively.

**Figure 4:**
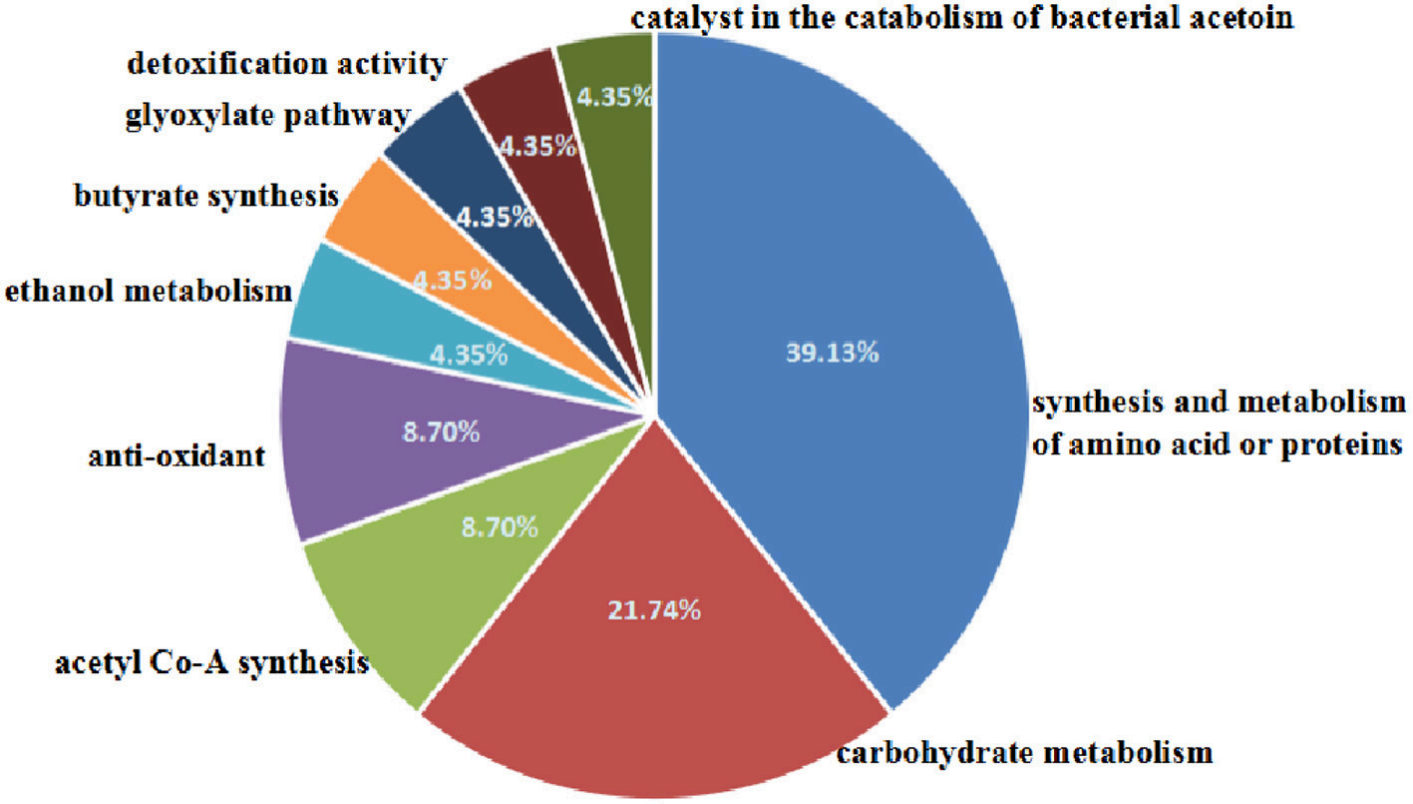
Distribution of the proteins spots identified among different classes by biological process.

The 2-D electrophoresis of the total bacterial proteins was shown in Figure 3. Twenty five different spots were found out; only 23 kinds of proteins were identified by HPLC-MS/MS and database. Among them, 18 expressed down-regulated proteins and 5 expressed up-regulated proteins were reported in Table 2. The distribution of the identified proteins was shown in Figure 4. Proteins with a high frequency were synthesis and metabolism of amino acid or proteins (39.13%) and carbohydrate metabolism (21.74%), anti-oxidant (8.70%), and acetyl Co-A synthesis (8.70%). Proteins with a low frequency were involved in the catalyst of catabolism of bacterial acetoin (4.35%), ethanol metabolism (4.35%), glyoxylate pathway (4.35%), butyrate synthesis (4.35%), and detoxification activity (4.35%).

**Table 2:** The different protein spots between microwave-treated bacteria and the control identified by HLPC-MS/MS.

**Code**	**Accession No**.	**Protein name**	**MASCOT score**	**Peptides matched**	**PI**	**MW (kDa)**	**Sequence coverage**	**Fold changes**	**Function**
4216	gi|29898596	L-lactate dehydrogenase	648	7	5.25	34.962	30%	−6.33	Carbohydrate metabolism
4119	gi|29894389	Transaldolase	365	8	5.26	23.169	52%	−5.14	Carbohydrate metabolism
3317	gi|29898226	Malate dehydrogenase	559	8	5.12	33.491	43%	−9.66	Carbohydrate metabolism
2415	gi|29898927	Fructose-bisphosphate aldolase	288	9	5	30.825	47%	+14.78	Carbohydrate metabolism
1122	gi|39932246	Triosephosphate isomerase	42	5	4.94	26.68	28%	+5.91	Carbohydrate metabolism
4618	gi|29895788	Formate-tetrahydrofolate ligase	861	9	5.37	60.754	27%	−3.28	Synthesis and metabolism of amino acid or proteins
4313	gi|29897470	Protein Translation Elongation Factor Ts (EF-Ts)	630	8	5.25	32.537	30%	−4.55	Synthesis and metabolism of amino acid or proteins
118	gi|29894976	Cell envelope-bound metalloprotease (camelysin)	198	5	4.71	21.801	32%	−6	Synthesis and metabolism of amino acid or proteins
1527	gi|29898334	Aminoacyl-histidine dipeptidase	303	4	4.88	51.411	17%	+16.1	Synthesis and metabolism of amino acid or proteins
7413	gi|29894155	Arginine deiminase	1062	10	6.11	47.022	37%	−5.83	Synthesis and metabolism of amino acid or proteins
6522	gi|29897865	Glycine dehydrogenase,	616	7	5.66	49.652	20%	−6.36	Synthesis and metabolism of amino acid or proteins
6315	gi|29897520	Proline dipeptidase	559	4	5.87	40.012	24%	−4.72	Synthesis and metabolism of amino acid or proteins
5429	gi|29894348	2-amino-3-ketobutyrate coenzyme A ligase	390	6	5.51	43.207	21%	−12.47	Synthesis and metabolism of amino acid or proteins
4710	gi|29893849	Methionyl-tRNA synthetase	167	7	5.31	75.27	13%	−19.53	Synthesis and metabolism of amino acid or proteins
4117	gi|29899036	Superoxide dismutase (Mn)	708	7	5.37	24.098	67%	−6.83	Anti-oxidant
4115	gi|29897912	Superoxide dismutase (Mn)	576	6	5.34	24.738	55%	−6.14	Anti-oxidant
3512	gi|29897615	Dihydrolipoamide dehydrogenase	559	8	5.25	49.637	28%	−9.4	Acetyl Co-A synthesis
5515	gi|29896450	Dihydrolipoamide dehydrogenase	295	4	5.86	49.386	20%	+26.21	Acetyl Co-A synthesis
6603	gi|29896451	Dihydrolipoamide acetyltransferase	152	5	6.16	43.33	15%	+7.36	Catalyst in the catabolism of bacterial acetoin
4419	gi|29895905	Ethanol dehydrogenase	389	5	5.38	37.521	20%	−4.74	Ethanol metabolism
4317	gi|29895108	Glyoxylate reductase (NADP+)	455	7	5.35	35.457	35%	−8.47	Glyoxylate pathway
8206	gi|29897804	Phosphate butyryl transferase	262	6	5.8	31.636	37%	−12.73	Butyrate synthesis
6520	gi|29895141	Nitric oxide dioxygenase	192	5	5.7	45.087	22%	−12.31	Detoxification activity

*“−” and “+” indicated the down-regulated and up-regulated proteins, respectively*.

Many studies about microbial destruction by microwave treatment have been reported. Halpin et al. (2014) suggested that non-themal treatments for *Pseudomonas fluorescens* and *Escherichia coli* had a higher inactivation than that of the thermal treatments. The destruction of microorganisms by microwave at temperatures lower than the thermal destruction point has been observed (Woo et al., 2000). In particular, microwave-stressed cells of *S. aureus* exhibited a greater metabolic imbalance than conventionally heated cells. Microwave radiation in *Escherichia coli* and *Bacillus subtilis* suspensions resulted in a dramatic reduction of the viable counts as well as increases in the amounts of released DNA and protein (Woo et al., 2000).

Similarly, in this study, we found that microwave treatment destroyed cell membrane and resulted in the leakage of intracellular components, which could contribute to metabolic imbalance of *B. cereus*. Proteomics analysis further showed that microwave treatments resulted in the changes of 23 proteins which were related to disorder the function of carbohydrates metabolism, the synthesis and metabolism of proteins, manganese superoxide dismutase, and so on. The disruption of function promoted the metabolic imbalance of *B. cereus*.

#### Proteins involved in carbohydrates metabolism

Five proteins were differentially expressed before and after microwave treatment involved in carbohydrates metabolism (21.74% frequency, Figure 4): L-lactate dehydrogenase, transaldolase, malate dehydrogenase, fructose-biphosphate aldolase, and triosephosphate isomerase (Rajesh et al., 2011). L-lactate dehydrogenase is an enzyme that catalyzes the reversible interconversion of pyruvate and lactate. Transaldolase is an enzyme which links the pentose phosphate pathway to glycolysis. Malate dehydrogenase is an enzyme that reversibly catalyzes the oxidation of malate to oxaloacetate using the reduction of NAD^+^ to NADH. Fructose-bisphosphate aldolase is a key enzyme catalyzing the reversible conversion of fructose-1, 6-bisphosphate to glyceraldehydes-3-phosphate and dihydroxyacetone phosphate (Du et al., 2014). Triosephosphate isomerase is an enzyme that catalyzes the interconversion of the triose phosphate isomers between dihydroxyacetone phosphate and D-glyceraldehyde 3-phosphate. In our results, microwave treatment down-regulated L-lactate dehydrogenase, transaldolase and malate dehydrogenase and up-regulated fructose-biphosphate aldolase and triosephosphate isomerase. It indicated that microwave treatment disordered the function of carbohydrates metabolism of *B. cereus*.

#### Proteins involved in the synthesis and metabolism of amino acids or proteins

The relevant proteins involved in the synthesis and metabolism of amino acids or proteins (39.13% frequency, Figure 4) were comprised of formate-tetrahydrofolate ligase, elongation factor Ts (EF-Ts), cell envelope-bound metalloprotease, aminoacyl-histidine dipeptidase, proline dipeptidase, arginine deminase, glycine dehydrogenase, 2-amino-3-ketobutyrate coenzyme A ligase and methionyl-tRNA synthetase.

Formate-tetrahydrofolate ligase participates in glyoxylate and dicarboxylate metabolism. EF-Ts serves as the exchange of EF-Tu-bound nucleotides via an EF-Tu–EF-Ts complex. The cell envelope-bound metalloprotease has high proteolytic activities in their cell envelopes including typical p-nitroanilide and furylacroleyl substrates (Fricke et al., 2001). The arginine deiminase system protects bacterial cells against the damaging effects of acid environments (Casiano-Colón and Marquis, 1988). The aminoacyl-histidine dipeptidase protein is involved in the synthesis of amino acids such as alanine, aspartate, arginine, proline, and histidine (Soni et al., 2007). Proline dipeptidase is a serine protease that cleaves dipeptides off the N-terminus of proteins when the penultimate amino acid is a proline or an alanine (Chiravuri et al., 2000). Glycine dehydrogenase participates in glycine, serine and threonine metabolism (Zhang et al., 2012). 2-Amino-3-ketobutyrate CoA ligase is a pyridoxal phosphate dependent enzyme involved in the degradation pathway of threonine. Methionyl-tRNA synthetase plays an essential role in initiating translation by transferring Met to initiator tRNA (tRNAiMet; Kwon et al., 2011). In our results, microwave treatment almost down-regulated all of proteins involved in the synthesis and metabolism of amino acids or proteins except histidine dipeptidase. It indicated that microwave treatment inhibited the function of synthesis and metabolism of amino acids or proteins of *B. cereus*.

#### Proteins involved in anti-oxidant function

Mn-SOD are a kind of enzymes scavenging free radicals generated in biological metabolism effectively and protecting cells against oxidative damage (Lee et al., 2001). In our results, two kinds of SOD were down-regulated (8.7% frequency, Figure 4). It demonstrated that microwave treatment inhibited the function of eliminating intracellular free radicals.

#### Other proteins

Alcohol dehydrogenase involves in ethanol metabolism to catalyze the reversible oxidation of primary or secondary alcohols to aldehydes or ketones (Atsumi et al., 2010). Dihydrolipoamide dehydrogenase are a kind of enzymes involved in acetyl Co-A synthesis (Janssen et al., 2006). Glyoxylate reductases involves in glyoxylate pathway to catalyze the NADH-linked reduction of glyoxylate to glycolate. Dihydrolipoamide acetyltransferase is part of the pyruvate dehydrogenase complex together with pyruvate dehydrogenase and dihydrolipoyl dehydrogenase involved in the catabolism of bacterial acetoin (Nemeria et al., 2002). Phosphate butyryl transferase involves in butyrate synthesis to catalyze the conversion of butyryl-CoA to butyrate. Nitric oxide dioxygenase is an enzyme with detoxification activity to catalyze the conversion of nitric oxide to nitrate (Forrester and Foster, 2012).

In our results, the proteins involved in ethanol metabolism (4.35% frequency, Figure 4), glyoxylate pathway (4.35% frequency, Figure 4), butyrate synthesis (4.35% frequency, Figure 4), and detoxification activity (4.35% frequency, Figure 4) were down-regulated. One kind of dihydrolipoamide dehydrogenase was down-regulated, while another was up-regulated. In addition, dihydrolipoamide acetyltransferase was up-regulated. It demonstrated that microwave treatment inhibited the function of ethanol metabolism, glyoxylate pathway, butyrate synthesis and detoxification activity, disordered acetyl Co-A synthesis and accelerated the catabolism of acetoin.

## Conclusion

The microwave treatment inactivate the *B. cereus* by inducing the clean of bacterial nuclear chromatin disrupting the cell membrane, increasing permeability of membrane and dysfunction of biological processes of *B. cereus*. Twenty-three differential proteins were closely related to dysfunction of biological processes and the metabolic imbalance of *B. cereus*. Further study is needed to elaborate how differential proteins result in the metabolic imbalance of *B. cereus*. This paper provides a reference for meat products industry to guarantee their safety, especially kill the *B. cereus* using microwave sterilization.

## Author contributions

J-XC and FW doing the experiment and the writing of manuscript. XL edited the manuscript. Y-YS and YW provided some help to the experiment. C-RO and X-FS participated in the revision of manuscript. D-DP participated in the writing of manuscripts.

### Conflict of interest statement

The authors declare that the research was conducted in the absence of any commercial or financial relationships that could be construed as a potential conflict of interest.
